# The value of NLR, PLR, PCT, and D-D levels in assessing the severity of hyperlipidemic acute pancreatitis

**DOI:** 10.3389/fmed.2025.1561255

**Published:** 2025-05-07

**Authors:** Lili Pan, Jinliang Xiao, Lijuan Fan

**Affiliations:** ^1^Department of Gastroenterology, Jining No. 1 People's Hospital, Jining, Shandong, China; ^2^The Department of Cardiology, Weihai City Central Hospital, Weihai, Shandong, China

**Keywords:** HLAP, platelets, neutrophils, lymphocytes, PCT, D-D, severity assessment

## Abstract

**Background:**

The incidence of hyperlipidemic acute pancreatitis (HLAP) has been increasing yearly, presenting a younger demographic and high mortality rates. Early assessment of severity is essential for improving treatment strategies and outcomes. Various serum biomarkers, including neutrophil-to-lymphocyte ratio (NLR) and platelet-to-lymphocyte ratio (PLR), have shown prognostic value in inflammatory conditions.

**Objective:**

This study aims to evaluate NLR, PLR, procalcitonin (PCT), and D-dimer (D-D) as biomarkers in assessing HLAP severity, identify independent predictors factors for moderate-to-severe HLAP, and provide evidence for tailored therapeutic interventions.

**Methods:**

A retrospective analysis was conducted on 145 HLAP patients admitted between mid-2021 and mid-2023 at Jining No. 1 People's Hospital. Patients were categorized into mild HLAP and moderate-to-severe HLAP groups based on severity. Clinical and biochemical data were analyzed using SPSS 26.0, and the diagnostic efficacy of NLR, PLR, PCT, and D-D was evaluated via Receiver Operating Characteristic (ROC) curve.

**Results:**

Elevated levels of NLR, PLR, PCT, and D-D were identified as independent predictors for moderate-to-severe HLAP. The combined ROC curve for these biomarkers yielded an area under the curve (AUC) of 0.898 (95% CI: 0.841–0.956), with sensitivity and specificity of 0.818 and 0.892, respectively, outperforming individual biomarkers.

**Conclusions:**

The combined assessment of NLR, PLR, PCT, and D-D is a simple, efficient, and cost-effective method for early HLAP severity evaluation. Further large-scale studies are warranted to validate these findings.

## 1 Introduction

The incidence of hyperlipidemic acute pancreatitis (HLAP) is rising globally, with an increasing number of cases reported among younger individuals. This trend may be linked to lifestyle changes, including poor dietary habits and rising rates of hyperlipidemia. HLAP is associated with a high risk of complications, substantial mortality, and frequent recurrence, making early and accurate assessment of disease severity essential for guiding timely clinical interventions and improving patient outcomes (1, 2).

Recent studies have shown that several serum biomarkers—including the neutrophil-to-lymphocyte ratio (NLR) (3), platelet-to-lymphocyte ratio (PLR) (4), procalcitonin (PCT) (5), and D-dimer (D-D) (6)—have demonstrated considerable value in monitoring inflammation, predicting complications, and assessing disease severity in acute pancreatitis (AP). Among them, NLR and PLR reflect systemic inflammation and immune status, PCT is highly sensitive to bacterial infection and sepsis, and D-D indicates a hypercoagulable state, often associated with disease severity (4, 5, 7–9). Based on these characteristics, this study selected NLR, PLR, PCT, and D-D as key biomarkers to evaluate their predictive value in hyperlipidemic acute pancreatitis. Although these markers have been individually investigated in AP, their combined application in HLAP remains largely unexplored. Recent literature supports the utility of multi-biomarker models in improving early risk stratification and severity prediction (4, 10).

Compared to previous studies that primarily focused on single biomarkers or general acute pancreatitis (AP) populations, this study presents a novel approach by developing and validating a four-biomarker assessment model tailored specifically for hyperlipidemic acute pancreatitis (HLAP). The model integrates three critical pathological dimensions of HLAP—systemic inflammation, coagulation dysfunction, and infection risk—through the combination of NLR, PLR, PCT, and D-D, all of which are routinely available laboratory indicators. This strategy facilitates rapid and accurate early risk stratification and demonstrates favorable feasibility, cost-effectiveness, and clinical applicability. While prior studies have largely investigated these biomarkers separately or in broader AP contexts, our study comprehensively evaluates their combined predictive performance in HLAP, helping to fill an existing gap in the literature. Additionally, we have identified independent predictors for moderate to severe HLAP, and showed that this biomarker model improves diagnostic accuracy, supports individualized treatment planning, and minimizes unnecessary testing and resource use, thereby contributing to enhanced clinical outcomes.

## 2 Materials and methods

### 2.1 Study design

We retrospectively analyzed 145 patients diagnosed with hyperlipidemic acute pancreatitis (HLAP) who were admitted to the Department of Gastroenterology at Jining No. 1 People's Hospital between the second half of 2021 and the second half of 2023. Inclusion Criteria: patients meeting the following criteria were included in the study: acute pancreatitis (AP) diagnosis: diagnosed based on the 2012 Atlanta Consensus criteria. AP was diagnosed if at least two of the following criteria were met: (1) acute upper abdominal pain typical of AP, often radiating to the back and associated with nausea or vomiting; (2) serum amylase and/or lipase levels ≥ three times the upper normal limit; (3) characteristic imaging findings (e.g., pancreatic edema or peripancreatic fluid collection on CT or MRI. HLAP was diagnosed when AP criteria were met along with one of the following: (1) serum triglyceride (TG) ≥ 11.3 mmol/L; or (2) TG between 5.65–11.3 mmol/L with a milky serum appearance, after excluding other causes of AP (e.g., biliary disease, drug use, alcohol abuse, or pancreatic duct abnormalities). Signed informed consent for participation in the study. Exclusion Criteria: patients were excluded under the following conditions: (1) Received treatment for AP prior to admission. (2) Admission occurred more than 24 h after AP onset. (3) Concurrent infections of other organs upon onset (e.g., pneumonia, cholecystitis, or appendicitis). (4) Incomplete clinical data or lack of blood samples collected within 24 h of admission.

Patients were categorized into two groups based on the revised Atlanta classification: a mild HLAP group (*n* = 75) and a moderate-to-severe HLAP group (*n* = 70). The study protocol was approved by the Ethics Review Committee of Jining No. 1 People's Hospital.

All patients were managed according to standardized HLAP treatment protocols based on national guidelines (11), including early fasting, aggressive fluid resuscitation, and low-molecular-weight heparin (LMWH) administration within the first 24 h of admission. Antibiotics were reserved for cases with confirmed infection.

### 2.2 Variables and definitions

Baseline data collected included patient age, body mass index (BMI), and the frequency of recurrence within 1 year. Vital signs, including heart rate, systolic blood pressure, diastolic blood pressure, body temperature, and respiratory rate, were also recorded. Laboratory data included complete blood count (neutrophils, lymphocytes, platelets), PCT, and biochemical markers (triglycerides, cholesterol, low-density lipoprotein, high-density lipoprotein, alanine aminotransferase, aspartate aminotransferase, gamma-glutamyl transferase, and total bilirubin). D-dimer (D-D) levels were measured, and the neutrophil-to-lymphocyte ratio (NLR) and platelet-to-lymphocyte ratio (PLR) were calculated.

### 2.3 Statistical analysis

Data were analyzed using SPSS 26.0 software. A normality test was performed for all quantitative data. Normally distributed data were presented as mean ± standard deviation (SD) and compared using independent-samples *t*-tests. Non-normally distributed data were expressed as medians (interquartile range, IQR) and analyzed using non-parametric tests (*Z*-tests). Statistical significance was defined as *P* < 0.05. For variables with significant differences, multivariate logistic regression analysis was performed to identify independent risk factors for moderate-to-severe HLAP. Receiver operating characteristic (ROC) curves were used to evaluate the diagnostic performance of NLR, PLR, PCT, and D-D levels, both individually and in combination, for moderate-to-severe HLAP. The cutoff values were determined using the Youden Index, and the area under the curve (AUC), sensitivity, and specificity were calculated.

## 3 Results

### 3.1 Patient characteristics

In the two patient groups, the moderate-to-severe HLAP group exhibited a significantly higher recurrence rate within 1 year compared to the mild HLAP group (*P* < 0.05). Age and BMI did not differ significantly between the two groups (*P* > 0.05; Table 1).

**Table 1:** Baseline characteristics comparison between the two groups.

**Variable**	**Mild HLAP group (*n* = 75)**	**Moderate/severe HLAP group (*n* = 70)**	***T/Z***	***P-value***
Age (years)	32.86 ± 6.98	30.85 ± 6.22	1.65	0.10
BMI (kg/m^2^)	27.16 ± 4.23	28.10 ± 5.15	−1.09	0.28
Number of recurrences within 1 year (times/year)	1.03 ± 0.79	1.76 ± 1.09	−4.15	<0.01^*^

* Indicates statistical significance (*P* < 0.05).

### 3.2 Comparison of vital signs between the two groups

Heart rate, temperature, diastolic pressure, and respiratory rate were comparable between groups (*P* > 0.05). Systolic blood pressure was significantly higher in the moderate-to-severe group (*P* < 0.05; Table 2).

**Table 2:** Comparison of vital signs between the two groups.

**Variable**	**Mild HLAP group (*n* = 75)**	**Moderate/severe HLAP group (*n* = 70)**	***T/Z***	***P-value***
Heart rate (times/min)	87.28 ± 16.77	87.24 ± 16.74	0.13	0.99
Systolic blood pressure (mmHg)	131.20 ± 18.61	148.69 ± 24.08	−4.39	<0.01^*^
Diastolic blood pressure (mmHg)	85.08 ± 12.98	81.58 ± 11.93	1.53	0.13
Body temperature (°C)	36.53 ± 0.28	36.60 ± 0.34	−1.38	0.17
Respiratory rate (times/min)	19.06 ± 2.21	18.38 ± 3.03	1.42	0.16

* Indicates statistical significance (*P* < 0.05).

### 3.3 Comparison of hematological, infection, and coagulation indicators between the two groups

NLR, PLR, PCT, and D-D levels were significantly elevated in the moderate-to-severe group (*P* < 0.05; Table 3).

**Table 3:** Comparison of hematological, infection, and coagulation indicators between the two groups.

**Variable**	**Mild HLAP group (*n* = 75)**	**Moderate/severe HLAP group (*n* = 70)**	***T/Z***	***P-value***
NLR	8.25 ± 7.93	15.38 ± 6.49	0.13	<0.01^*^
PLR	126.92 ± 84.86	306.67 ± 143.25	−4.39	<0.01^*^
D-D (mg/L)	1.26 ± 0.96	2.62 ± 1.25	1.53	<0.01^*^
PCT (pg/mL)	0.75 ± 0.87	1.87 ± 0.71	1.42	<0.01^*^

* Indicates statistical significance (*P* < 0.05).

### 3.4 Comparison of biochemical indicators between the two groups

Triglyceride and cholesterol levels were also significantly higher in the moderate-to-severe group (*P* < 0.05; Table 4).

**Table 4:** Comparison of biochemical indicators between the two groups.

**Variable**	**Mild HLAP group (*n* = 75)**	**Moderate/severe HLAP group (*n* = 70)**	***T/Z***	***P-value***
Triglyceride (mmol/L)	7.98 ± 2.55	12.51 ± 8.89	−3.65	<0.01^*^
Cholesterol (mmol/L)	6.90 ± 2.53	9.99 ± 5.86	−3.63	<0.01^*^
LDH (mmol/L)	2.41 ± 1.23	2.69 ± 1.06	−1.29	0.20
HDL (mmol/L)	0.91 ± 0.46	1.32 ± 1.71	−1.73	0.09
ALT (μ/L)	28.93 ± 18.98	23.30 ± 17.27	1.69	0.09
AST (μ/L)	23.57 ± 15.56	28.66 ± 18.65	−1.63	0.11
GGT (μ/L)	28.93 ± 18.98	23.30 ± 17.27	1.69	0.09
TBIL (μmol/L)	19.37 ± 8.02	20.80 ± 12.14	−0.77	0.44

* Indicates statistical significance (*P* < 0.05).

### 3.5 Multivariate logistic regression analysis of the moderate-to-severe HLAP group

Multivariate analysis identified NLR (OR = 1.225), PLR (OR = 1.008), D-D (OR = 2.535), and PCT (OR = 4.033) as independent predictors of moderate-to-severe HLAP (*P* < 0.05; Table 5).

**Table 5:** Multivariate logistic regression analysis of independent predictors for moderate-to-severe HLAP.

**Variable**	***B***	***SE***	***Waldx^2^***	***P***	***OR***	***95%Cl***
NLR	0.203	0.064	10.013	0.002^*^	1.225	1.080–1.389
PLR	0.008	0.004	3.984	0.046^*^	1.008	1.000–1.016
D-D	0.930	0.436	4.559	0.033^*^	2.535	1.079–5.952
PCT	1.395	0.525	7.050	0.008^*^	4.033	1.441–11.291
Number of recurrences within a year	0.354	0.415	0.728	0.394	1.425	0.632–3.216
Triglyceride	0.273	0.143	3.677	0.055	1.314	0.994–1.738
Cholesterol	0.033	0.141	0.055	0.814	1.034	0.785–1.362
Systolic blood pressure	0.005	0.019	0.062	0.803	1.005	0.968–1.042

* Indicates statistical significance (*P* < 0.05).

### 3.6 ROC curve analysis of NLR, PLR, D-D, PCT, and their combination

**Figure 1:**
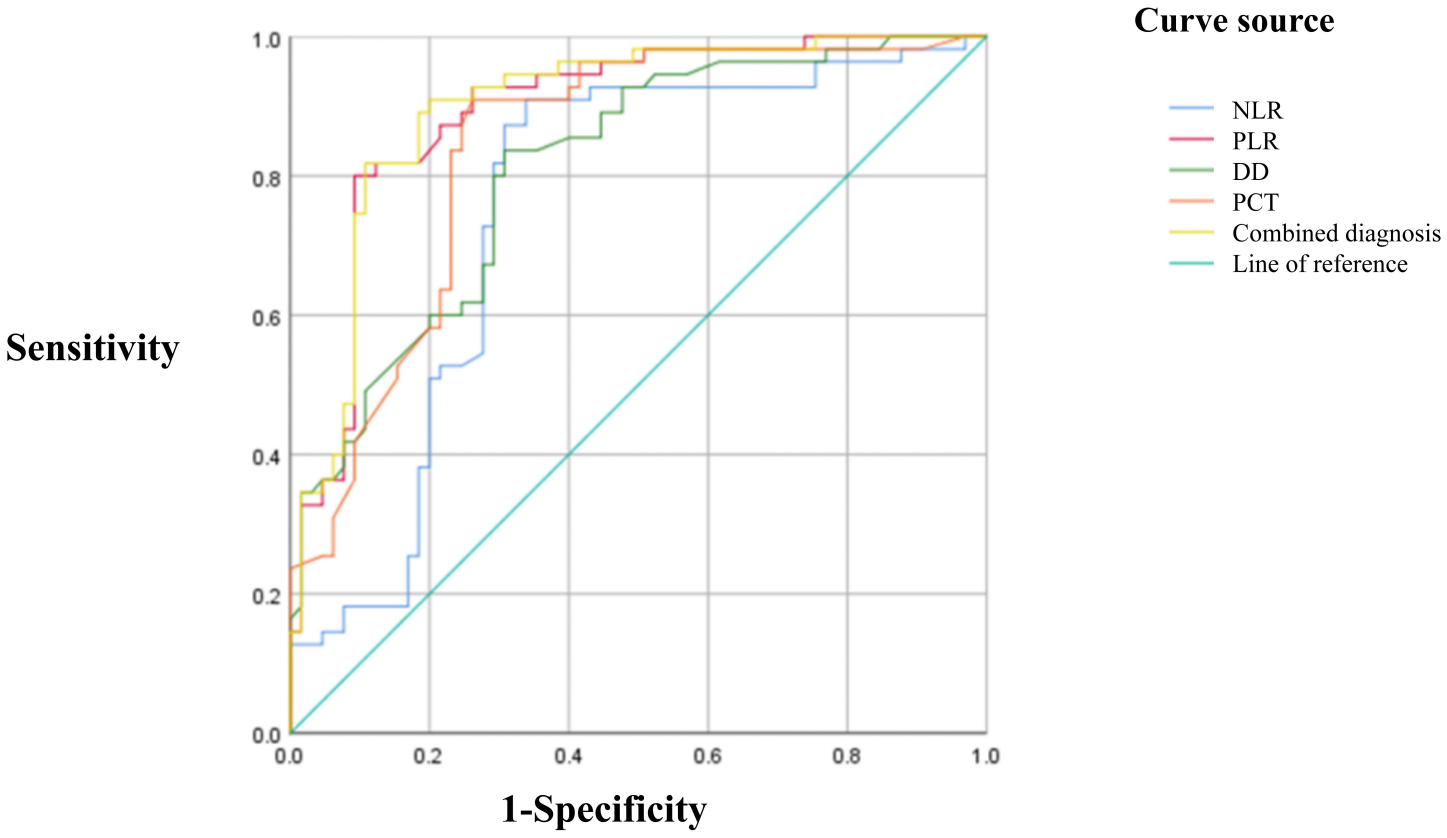
ROC curves for NLR, PLR, D-D, PCT, and their combination.

ROC curve analysis revealed AUCs of 0.752 for NLR, 0.892 for PLR, 0.809 for D-D, 0.839 for PCT, and 0.898 for the combined model. The corresponding Youden indices were 0.571, 0.708, 0.528, 0.647, and 0.710, with optimal cutoff values of 7.48, 209.66, 1.535, 1.09, and 207.92, respectively. Sensitivities were 0.909, 0.800, 0.836, 0.909, and 0.818; specificities were 0.662, 0.908, 0.692, 0.738, and 0.892, respectively. The combined use of NLR, PLR, D-D, and PCT achieved the highest AUC and demonstrated superior predictive performance compared to individual markers (Table 6; Figure 1).

**Table 6:** ROC curve analysis of NLR, PLR, D-D, PCT, and their combination.

**Variable**	***AUC***	***P***	**95%CI**	**Youden**	***Cut-off***	**Sensitivity**	**Specificity**
NLR	0.752	<0.001^*^	0.661–0.843	0.571	7.48	0.909	0.662
PLR	0.892	<0.001^*^	0.833–0.951	0.708	209.66	0.8	0.908
D-D	0.809	<0.001^*^	0.733–0.885	0.528	1.535	0.836	0.692
PCT	0.839	<0.001^*^	0.767–0.911	0.647	1.09	0.909	0.738
Fourfold union	0.898	<0.001^*^	0.841–0.956	0.71	207.92	0.818	0.892

* Indicates statistical significance (*P* < 0.05).

## 4 Discussion

Hyperlipidemic acute pancreatitis (HLAP) is a clinically diverse and life-threatening condition with an increasing incidence and mortality rate. Early identification and assessment of HLAP severity are critical for guiding treatment strategies and improving patient outcomes.

Although NLR is not a specific diagnostic marker for AP, elevated levels have been associated with disease severity and prognosis (12). High NLR levels may indicate a more pronounced inflammatory response and immune dysregulation. During AP, inflammation and tissue injury in the pancreas can trigger systemic inflammatory responses and alterations in immune function (7, 13). During AP, neutrophils increase while lymphocytes decrease, reflecting immune dysregulation (7, 13). The calculation of NLR reflects changes in inflammation and immune status.

In HLAP, tissues are damaged by inflammatory cytokines, proteases, and oxidative stress, primarily driven by inflammation. Neutrophils, which secrete these damaging substances, further accelerate the inflammatory process (14). Inflammatory cytokines can stimulate platelet production, contributing to elevated PLR (15). The immune system, including lymphocytes and the lymphatic system, plays a crucial role in modulating and slowing the inflammatory process. Uncontrolled inflammation in HLAP can lead to lymphocyte apoptosis and redistribution, reducing immune cells, particularly lymphocytes (16). The combination of systemic inflammatory response syndrome (SIRS) and multi-organ failure (MOF) accelerates the progression to moderate-to-severe HLAP, with inflammatory mediators driving SIRS (17). While these markers have demonstrated utility in AP, limited data exist regarding their application in HLAP. This study aims to determine the predictive value of these biomarkers in evaluating HLAP severity.

Procalcitonin (PCT) plays a critical role in immune responses, with a half-life of 24 h. PCT helps diagnose infection and monitor antibiotic response. Its broad-spectrum antimicrobial properties and favorable pharmacokinetics make it a valuable clinical tool. Under PCT-guided protocols, the use of antibiotics can reduce side effects and improve clinical outcomes. PCT levels also increase in conditions such as trauma, burns, heart failure, pancreatitis, and infections. Inflammatory cytokines (e.g., IL-6, TNF-α) in the tumor microenvironment stimulate PCT production, rapidly elevating its blood concentration. Studies have shown that PCT is more sensitive and specific than C-reactive protein (CRP) in assessing AP prognosis and disease severity (5). Yin et al. (18) also observed significantly higher PCT levels in moderate-to-severe AP patients compared to those with mild AP, further supporting its role as a promising serum biomarker for evaluating AP severity.

Elevated D-dimer (D-D) levels in AP patients may indicate systemic inflammation and thrombosis, correlating with more severe disease and poor outcomes (6, 19). AP often triggers coagulation processes early in its course (20), leading to pancreatic necrosis, bleeding, and shock. As a fibrin degradation product, D-D is a sensitive marker of thrombosis and is used in conditions like pulmonary embolism (PE), myocardial infarction (MI), and disseminated intravascular coagulation (DIC). Studies indicate that higher D-D levels are associated with severe AP and adverse outcomes (6). Recent research highlights significant differences in D-D levels across AP severity groups, confirming its relevance in evaluating HLAP severity (21).

This study analyzed independent predictor's factors for moderate-to-severe HLAP, focusing on the utility of NLR, PLR, PCT, and D-D in improving early diagnostic accuracy and guiding tailored treatment strategies. These biomarkers reflect distinct pathophysiological processes such as systemic inflammation, coagulation activation, and infection. Their elevation may collectively indicate systemic dysregulation in severe HLAP, serving as predictive indicators rather than direct causal factors. Unlike traditional scoring systems such as Ranson and BISAP, which require multiple clinical parameters and take up to 48 h to complete, this four-biomarker model offers a rapid, cost-effective, and accessible method for early HLAP risk stratification. The combination of NLR, PLR, PCT, and D-D not only improves predictive accuracy but also captures several key pathophysiological pathways—including systemic inflammation, immune dysregulation, thrombosis, and infection. This is particularly valuable in HLAP, which tends to progress rapidly and affects younger patients without significant comorbidities. To our knowledge, this is the first study to systematically evaluate this specific combination in HLAP, providing both methodological novelty and practical clinical value. These biomarkers can aid in timely identification of disease progression, improving patient prognosis, and reducing unnecessary diagnostic procedures to alleviate economic burdens. The combined ROC curve for NLR, PLR, PCT, and D-D yielded an AUC of 0.898 (95% CI: 0.841–0.956), with sensitivity and specificity of 0.818 and 0.892, respectively. This combined approach outperformed individual markers in predicting HLAP severity. The combination of NLR, PLR, D-D, and PCT is a simple, rapid, cost-effective, and practical method for assessing HLAP severity, warranting broader clinical application. Compared to Ranson's criteria, which require a 48 h data collection period and multiple biochemical tests, our biomarker panel enables risk stratification at the time of admission. Although the BISAP score has shown comparable predictive performance (22), it incorporates semi-subjective components such as altered mental status, which may be inconsistently assessed. In contrast, our approach is based entirely on objective, routinely measured laboratory parameters, offering timely, reproducible, and easily accessible results for early clinical decision-making.

Our NLR cutoff (7.48) is comparable to thresholds reported in general acute pancreatitis research (e.g., NLR >5.0 in Suppiah et al.) (3), but is slightly higher than typical values for non-hyperlipidemic AP. This may reflect the more intense inflammatory response characteristic of HLAP. The PLR cutoff (209.66) is consistent with previous studies that associated PLR >200 with poor prognosis in AP patients (4). For D-dimer, our identified cutoff (1.535 mg/L) is notably higher than those used in general AP populations likely due to HLAP-related hypercoagulability induced by elevated lipid levels (23). Although these thresholds are not yet included in current pancreatitis management guidelines, they are pathophysiologically relevant to HLAP and may help guide future research and clinical decision-making.

However, this study has limitations, including a relatively small sample size, which may restrict the generalizability of the findings. Larger, prospective studies are needed to validate the predictive value of these biomarkers in assessing HLAP severity.

## 5 Conclusion

This study shows that NLR, PLR, D-D, and PCT combined offer an objective and accessible tool for assessing HLAP severity. These biomarkers hold significant value for early intervention and improving clinical outcomes in HLAP patients. Further research is needed to confirm their utility and expand their clinical applications.

## Data Availability

The datasets presented in this study can be found in online repositories. The names of the repository/repositories and accession number(s) can be found in the article/supplementary material.

## References

[1] Szentesi A Parniczky A Vincze A Bajor J Godi S Sarlos P. Multiple hits in acute pancreatitis: components of metabolic syndrome synergize each other's deteriorating effects. Front Physiol. (2019) 10:1202. 10.3389/fphys.2019.01202 31620021 PMC6763590

[2] Zhou W Liu Q Wang Z Yao L Chen J Yang X. Analysis of the clinical profile and treatment efficiency of hyperlipidemic acute pancreatitis. Lipids Health Dis. (2024) 23:70. 10.1186/s12944-024-02057-5 38459563 PMC10921628

[3] Suppiah A Malde D Arab T Hamed M Allgar V Smith AM. The prognostic value of the neutrophil-lymphocyte ratio (NLR) in acute pancreatitis: identification of an optimal NLR. J Gastrointest Surg. (2013) 17:675–81. 10.1007/s11605-012-2121-1 23371356

[4] Kaplan M Ates I Oztas E Yuksel M Akpinar MY Coskun O. A new marker to determine prognosis of acute pancreatitis: PLR and NLR combination. J Med Biochem. (2018) 37:21–30. 10.1515/jomb-2017-0039 30581338 PMC6294107

[5] Rau BM Kemppainen EA Gumbs AA Buchler MW Wegscheider K Bassi C. Early assessment of pancreatic infections and overall prognosis in severe acute pancreatitis by procalcitonin (PCT): a prospective international multicenter study. Ann Surg. (2007) 245:745–54. 10.1097/01.sla.0000252443.22360.46 17457167 PMC1877072

[6] Yang N Zhang DL Hao JY. Coagulopathy and the prognostic potential of D-dimer in hyperlipidemia-induced acute pancreatitis. Hepatobiliary Pancreat Dis Int. (2015) 14:633–41. 10.1016/S1499-3872(15)60376-9 26663012

[7] Zhou H Mei X He X Lan T Guo S. Severity stratification and prognostic prediction of patients with acute pancreatitis at early phase: a retrospective study. Medicine. (2019) 98:e15275. 10.1097/MD.0000000000015275 31008971 PMC6494233

[8] Tarjan D Szalai E Lipp M Verboi M Koi T Eross B. Persistently high procalcitonin and C-Reactive protein are good predictors of infection in acute necrotizing pancreatitis: a systematic review and meta-analysis. Int J Mol Sci. (2024) 25:1273. 10.3390/ijms25021273 38279274 PMC10816999

[9] Xue E Shi Q Guo S Zhang X Liu C Qian B. Preexisting diabetes, serum calcium and D-dimer levels as predictable risk factors for pancreatic necrosis of patients with acute pancreatitis: a retrospective study. Expert Rev Gastroenterol Hepatol. (2022) 16:913–21. 10.1080/17474124.2022.2116314 36036225

[10] Poulsen VV Hadi A Werge MP Karstensen JG Novovic S. Circulating biomarkers involved in the development of and progression to chronic pancreatitis-a literature review. Biomolecules. (2024) 14:239. 10.3390/biom14020239 38397476 PMC10887223

[11] Chinese Pancreatic Surgery Association CSoSCMA. Guidelines for diagnosis and treatment of acute pancreatitis in China (2021). Zhonghua Wai Ke Za Zhi. (2021) 59:578–87. 10.3760/cma.j.cn112139-20210416-00172 34256457

[12] Park HS In SG Yoon HJ Lee WJ Woo SH Kim D. Predictive values of neutrophil-lymphocyte ratio as an early indicator for severe acute pancreatitis in the emergency department patients. J Lab Physicians. (2019) 11:259–64. 10.4103/JLP.JLP_82_19 31579249 PMC6771314

[13] Junare PR Debnath P Nair S Chandnani S Udgirkar S Thange R. Complete hemogram: simple and cost-effective in staging and predicting outcome in acute pancreatitis. Wien Klin Wochenschr. (2021) 133:661–8. 10.1007/s00508-021-01821-2 33620577

[14] Yang ZW Meng XX Xu P. Central role of neutrophil in the pathogenesis of severe acute pancreatitis. J Cell Mol Med. (2015) 19:2513–20. 10.1111/jcmm.12639 26249268 PMC4627557

[15] Couldwell G Machlus KR. Modulation of megakaryopoiesis and platelet production during inflammation. Thromb Res. (2019) 179:114–20. 10.1016/j.thromres.2019.05.008 31128560

[16] Li Y Zhao Y Feng L Guo R. Comparison of the prognostic values of inflammation markers in patients with acute pancreatitis: a retrospective cohort study. BMJ Open. (2017) 7:e013206. 10.1136/bmjopen-2016-013206 28348184 PMC5372142

[17] Zerem E. Treatment of severe acute pancreatitis and its complications. World J Gastroenterol. (2014) 20:13879–92. 10.3748/wjg.v20.i38.13879 25320523 PMC4194569

[18] Yin Y Wei N Zheng Z Liang H. Relationship between apoA-I, chemerin, procalcitonin and severity of hyperlipidaemia-induced acute pancreatitis. J Pak Med Assoc. (2022) 72:1201–4. 10.47391/JPMA.3700 35751336

[19] Radenkovic D Bajec D Karamarkovic A Stefanovic B Milic N Ignjatovic S. Disorders of hemostasis during the surgical management of severe necrotizing pancreatitis. Pancreas. (2004) 29:152–6. 10.1097/00006676-200408000-00010 15257107

[20] Gomercic C Gelsi E Van Gysel D Frin AC Ouvrier D Tonohouan M. Assessment of D-Dimers for the early prediction of complications in acute pancreatitis. Pancreas. (2016) 45:980–5. 10.1097/MPA.0000000000000654 27253234

[21] Yang N Hao J Zhang D. Antithrombin III and D-dimer levels as indicators of disease severity in patients with hyperlipidaemic or biliary acute pancreatitis. J Int Med Res. (2017) 45:147–58. 10.1177/0300060516677929 28222624 PMC5536593

[22] Wu BU Johannes RS Sun X Tabak Y Conwell DL Banks PA. The early prediction of mortality in acute pancreatitis: a large population-based study. Gut. (2008) 57:1698–703. 10.1136/gut.2008.152702 18519429

[23] Zhang GQ Wang G Li L Hu JS Ji L Li YL. Plasma D-Dimer level is an early predictor of severity of acute pancreatitis based on 2012 atlanta classification. Med Sci Monit. (2019) 25:9019–27. 10.12659/MSM.918311 31774737 PMC6898981

